# Enhanced rhLCV production in lymphoblastoid cell lines derived from rhLCV-infected cynomolgus macaque PBMCs

**DOI:** 10.1128/jvi.01821-25

**Published:** 2026-01-27

**Authors:** Ling Zhong, Yanran Luo, Wanlin Zhang, Qingbing Zheng, Xinyu Zhang, Xiaoping Ye, Qisheng Feng, Yi-Xin Chen, Xiao Zhang, Miao Xu

**Affiliations:** 1 State Key Laboratory of Oncology in South China, Collaborative Innovation Center for Cancer Medicine, Guangdong Key Laboratory of Nasopharyngeal Carcinoma Diagnosis and Therapy, Sun Yat-sen University Cancer Center 71067 , Guangzhou, China; 2 State Key Laboratory of Vaccines for Infectious Diseases, State Key Laboratory of Molecular Vaccinology and Molecular Diagnostics, Collaborative Innovation Center of Biologic Products, National Innovation Platform for Industry-Education Integration in Vaccine Research, Research Unit of Frontier Technology of Structural Vaccinology of the Chinese Academy of Medical Sciences, Xiang An Biomedicine Laboratory, School of Life Sciences, School of Public Health, Xiamen University 12466, Xiamen, China; 3 College of Pharmacy, Chongqing Medical University 12550, Chongqing, China; University of Toronto, Toronto, Ontario, Canada

**Keywords:** EBV, rhLCV production, *Macaca fascicularis*, lymphoblastoid cell line, cross-neutralization

## Abstract

Epstein-Barr virus (EBV) infects more than 90% of adults worldwide and causes a range of diseases, including multiple malignancies and autoimmune disorders. However, due to a host range restriction, EBV cannot infect commonly used experimental animals, posing a significant obstacle to developing EBV-specific prophylactic and therapeutic agents. Rhesus lymphocryptovirus (rhLCV), an ortholog of EBV, naturally infects rhesus macaques, which is a surrogate model for EBV research. In this study, we demonstrate that cynomolgus macaque (Macaca fascicularis), a primate closely related to rhesus macaque, is susceptible to rhLCV infection. rhLCV can immortalize B cells of cynomolgus macaques to develop cy-LCLs. We developed a high rhLCV-producing cy-LCL cell line, LCL111, and optimized the induction conditions to increase viral production, surpassing the original rhLCV producer LCL8664. Importantly, EBV gHgL-specific monoclonal antibody (mAb) AMMO1 and gB-specific mAb 3A5 can cross-react with rhLCV proteins and block the formation of cy-LCLs. Overall, we established an efficient rhLCV-producing cell line, and rhLCV infection of cynomolgus macaques represents a promising alternative surrogate model for efficiency evaluation of EBV vaccines and mAbs.

**IMPORTANCE:**

Epstein-Barr virus (EBV) naturally infects only humans, creating a major barrier to evaluating the efficiency of vaccines and therapies *in vivo*. As an EBV ortholog, rhesus lymphocryptovirus (rhLCV) offers a biologically relevant surrogate system. However, its application has been primarily limited to rhesus macaques. Here, we demonstrate that cynomolgus macaque lymphocytes are also susceptible to rhLCV *in vitro,* and the newly transformed cy-LCL111 shows superior and sustained rhLCV production ability. rhLCV infection of cynomolgus macaque lymphocytes can be efficiently neutralized by anti-EBV gH/gL nAbs AMMO1 and anti-EBV gB mAbs 3A5, highlighting the potential of cynomolgus macaques as an *in vivo* model to assess anti-EBV mAb and vaccine efficacy. Our findings support the use of cynomolgus macaques as an additional model for EBV research and offer a useful platform for evaluating EBV-specific prophylactic or therapeutic strategies.

## INTRODUCTION

Herpesviruses in the lymphocryptovirus (LCV) genus are identified in hominoids and old and new world non-human primates (NHP). LCVs share certain similarities, including immortalizing natural host B cells *in vitro* and establishing lifelong latency following primary infection (1, 2). Epstein-Barr virus (EBV), also known as human herpesvirus 4, infects more than 90% of adults worldwide. EBV primary infection in children is asymptomatic, while it induces infectious mononucleosis (IM) in adolescence (3, 4). EBV infection is also associated with various malignancies, including Hodgkin lymphoma, Burkitt lymphoma, nasopharyngeal carcinoma, gastric cancer, and NK/T lymphoma (3). In addition, EBV infection is linked to autoimmune diseases, such as multiple sclerosis, systemic lupus erythematosus, and rheumatoid arthritis (5). Although EBV infection causes heavy disease burdens, no prophylactic or therapeutic agent is available now.

Due to the host range restriction, a lack of animal models hinders the development of EBV-specific vaccines and drugs. Humanized mice and NHPs are widely used to evaluate the efficacy of EBV vaccine candidates. EBV infection and lymphoma formation can be observed in humanized mice under different challenge doses (6–8). Nevertheless, humanized mice are more suitable for evaluating the passive protective effect resulting from their immune systems remaining incomplete (5). The infection route in humanized mice, intravenous administration, is also distinct from the natural saliva transmission route of EBV infection. Among NHPs, EBV can infect cotton-top tamarins and common marmosets *in vivo*. However, these species are now essentially unavailable for research use due to ethical restrictions and limited resources (5, 9, 10). Moreover, it is reported that EBV can transform B cells from owl monkeys, chimpanzees, gibbons, and squirrel monkeys *in vitro* (11–15). However, whether EBV can infect these NHPs *in vivo* needs to be further determined. Rhesus lymphocryptovirus (rhLCV) infection of rhesus macaques is a promising surrogate model for EBV-related studies. The rhLCV genome was the first sequenced non-human LCV genome, lytic proteins of which are well conserved with EBV (1). Importantly, rhLCV can infect rhesus macaques via the oral route, which is similar to the natural infection route of EBV in humans (16, 17).

Experimental infection with rhLCV in rhesus macaques can result in either an asymptomatic persistent latent infection or an IM-like syndrome in immunocompetent animals (18). Moreover, humoral immune response to rhLCV glycoproteins, early proteins, and immediate early proteins was detected in rhLCV-infected rhesus macaques (19). In addition, rhLCV latent antigen-specific CD8^+^ T cells were detected in rhesus macaques persistently infected by rhLCV, resembling the immune response observed in EBV-infected adults (20). Hence, rhLCV infection of rhesus macaques is a promising surrogate model for EBV studies because of its high similarity to EBV in biology, genetics, and pathogenicity, as well as similarities in immune responses between humans and NHPs (2, 21–24).

rhLCV infection of rhesus macaques has been used to evaluate the protective efficiency of neutralizing monoclonal antibodies (nAbs) and vaccines against EBV. The protective efficiency of EBV gHgL-specific nAbs, including AMMO1 and E1D1, was determined in rhesus macaques infected by rhLCV (16, 17). Due to the low homology between rhLCV-gp350 and EBV-gp350, Wang et al. established an EBV gp350 chimeric rhLCV to evaluate the protective efficiency of EBV gp350-specific nAb 72A1 (25). Moreover, rhLCV infection of rhesus macaques is a potential model to evaluate the efficiency of EBV prophylactic vaccines and therapeutic vaccines. Rhesus macaques immunized with EBV gH/gL nanoparticle vaccines were protected from oral challenge of rhLCV (26). rhLCV EBNA-1-based therapeutic vaccine immunization expanded EBNA-1-specific T cells in rhesus macaques (27). To sum up, rhLCV infection of rhesus macaques is becoming a promising model for EBV vaccines and nAb studies. Hence, enhancing the production yield of rhLCV and standardizing methods of virus production and infection are urgently needed.

Given that the rhesus macaque supply falls short of research demand, there is an impetus need for a new available animal model. Cynomolgus macaques (*Macaca fascicularis*) are closely related to rhesus macaques. Previous studies have reported that rhLCV can infect peripheral blood mononuclear cells (PBMCs) from cynomolgus macaques *in vitro* (14). Despite this, whether the lymphoblastoid cell line (LCLs) transformed by exogenous LCV can be expanded and produce infectious exogenous LCV remains to be determined.

In this study, we successfully established three cy-LCLs via *in vitro* rhLCV infection of cynomolgus macaque PBMCs. We demonstrated that cy-LCL111 transformed by rhLCV exhibits high rhLCV production capacity. We developed a BALF-5-based qPCR approach to quantify rhLCV and demonstrated that at least 1.49 × 10¹⁰ genome copies of rhLCV were sufficient to induce 50% transformation of cynomolgus macaque PBMCs. In addition, EBV gB-specific nAb 3A5 and gHgL-specific nAb AMMO1 can inhibit rhLCV-induced cynomolgus macaque B-cell transformation. These results suggest that rhLCV infection of cynomolgus macaques may serve as a surrogate model for EBV-related research.

## RESULTS

### rhLCV production and characterization

**Fig 1:**
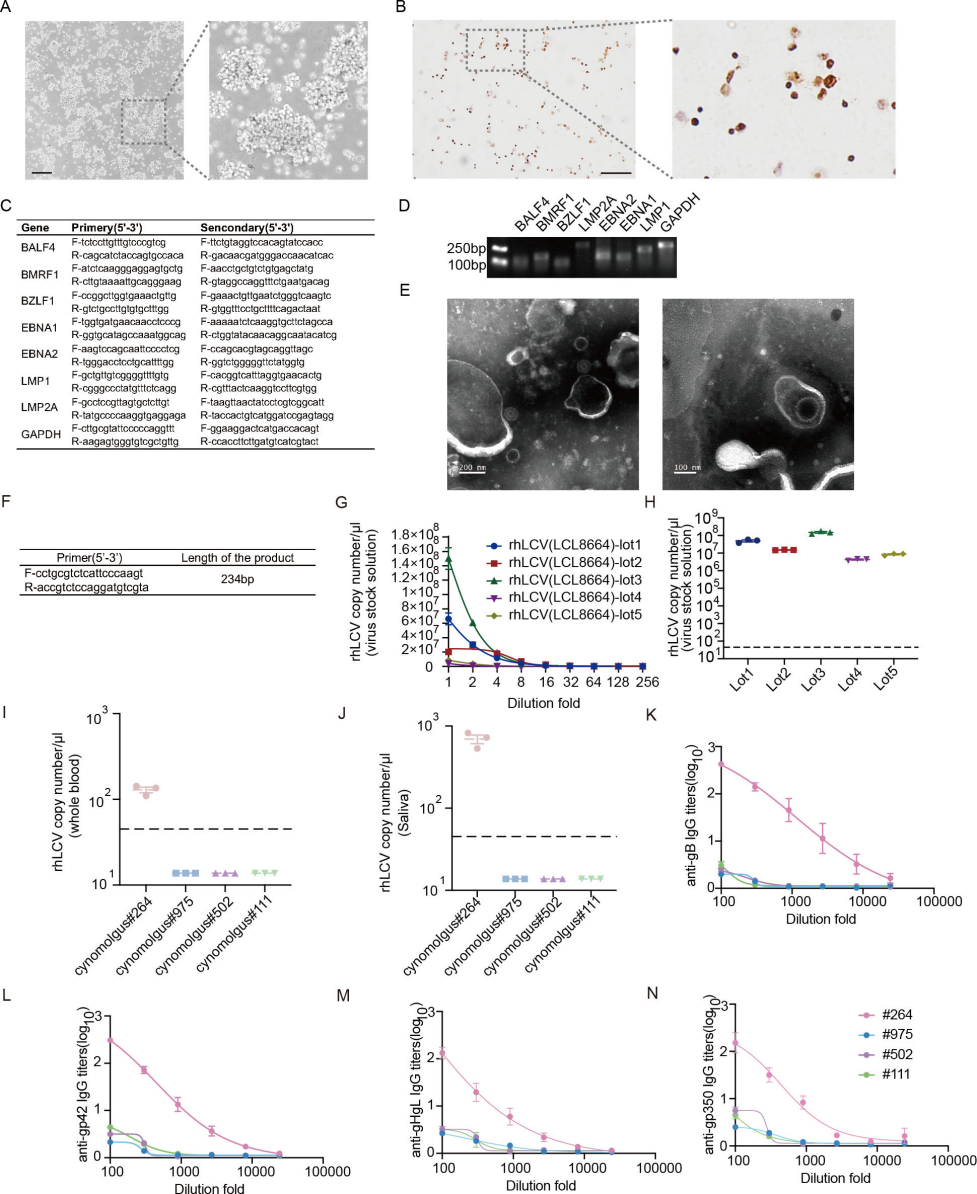
Development of a real-time qPCR-based method to determine the copy numbers of rhLCV. (**A**) Representative images of LCL8664 (scale bar = 500 μm). (**B**) Representative images of LCL8664 hybridized for EBER (scale bar = 50 μm). (**C**) Primers for nested PCR of rhLCV latent and lytic genes. (**D**) rhLCV gene expression in LCL8664. Primers used in this assay are shown in (**C**). (**E**) Negative staining TEM images of rhLCV virus generated by LCL8664 reveal the presence of viral capsids and viral genome (left panel: scale bar = 200 nm; right panel: scale bar = 100 nm). Due to the preparation procedure for TEM observation, the viral membrane structure was disrupted in the majority of viral particles, preventing its visualization. (**F**) Primers for real-time qPCR and the length of the PCR product. (**G**) rhLCV copy numbers per μL in the serially diluted different lots of viral stock solution after induced by 20 ng/mL TPA and 3 mM NaB for 12 h at a cell density of 3 × 10^6^ cells/mL. Primers are shown in (**F**). (**H**) rhLCV copy numbers per μL in the undiluted five lots of viral stock solution. The dashed line indicates the detection limit. Primers are shown in (**F**). (**I**) LCV copy numbers in the whole blood of cynomolgus macaques #264, #975, #502, and #111. The dashed line indicates the detection limit. Primers are shown in (**F**). (**J**) LCV copy numbers in the saliva of cynomolgus macaques #264, #975, #502, and #111. The dashed line indicates the detection limit. Primers are shown in (**F**). (**K**) Anti-EBV gB cross-reactive IgG titers in sera of four cynomolgus macaques. (**L**) Anti-EBV gp42 cross-reactive IgG titers in sera of four cynomolgus macaques. (**M**) Anti-EBV gHgL cross-reactive IgG titers in sera of four cynomolgus macaques. (**N**) Anti-EBV gp350 cross-reactive IgG titers in sera of four cynomolgus macaques. (**G–N**) Data points are shown as the mean ± SEM (*n* = 3).

The B lymphocyte cell line LCL8664 was derived from the peripheral blood of a 5-year-old male rhesus monkey diagnosed with lymphoma (22). LCL8664 is commonly used for rhLCV production at present, which grows aggregately like other LCLs (Fig. 1A). We confirmed that rhLCV-EBER can be detected by the EBER *in situ* hybridization kit, and almost all LCL8664 cells were rhLCV positive (Fig. 1B).

To further analyze the rhLCV gene expression status in LCL8664, we designed primers for nested PCR identification for rhLCV latent protein (EBNA1, EBNA2, LMP1, LMP2A), immediate early protein (BZLF1), early lytic protein (BMRF1), and late lytic protein BALF4 (Fig. 1C). The data showed that both latent and lytic phase genes were expressed in the cell line, indicating that rhLCV may be produced and released to cell culture supernatant with low titers, which was consistent with EBV-producing cell lines (Fig. 1D).

After concentrating the virus by ultracentrifugation, negative staining transmission electron microscopy (TEM) was used to visualize the rhLCV virion. The rhLCV virions detected by TEM exhibit a morphology similar to that of the EBV virion (28). The rhLCV viral capsids and internal genome were clearly observed (Fig. 1E). The TEM results further confirmed the successful production of rhLCV by induction of LCL8664. Nevertheless, the viral envelope and tegument were not detected in the majority of viral particles, likely due to membrane disruption caused by sample processing for TEM analysis (Fig. 1E). It is well known that negative staining, along with the associated dehydration and specimen drying steps, can cause disruption or collapse of the envelope and tegument. Cryo-EM or Cryo-ET, instead of negative-staining TEM, is generally used to visualize the intact membrane and tegument structures of herpesviruses (29).

Collectively, almost all LCL8664 cells are rhLCV positive, and the cell line is spontaneously lytic during cell culture. rhLCV was successfully produced for subsequent experiments. rhLCV is similar to EBV in morphology.

### rhLCV copy numbers determination

The commonly used approach for EBV titer quantification was based on flow cytometry since the green fluorescence protein (GFP) gene was inserted into the EBV genome. However, the rhLCV used in this study is a wild-type strain without a fluorescence protein. Previous studies have employed the LCL transformation assay to determine rhLCV titers (17, 26). The LCL transformation requires over 6 weeks, and multiple replicates are needed for each viral dilution. Hence, a less time-consuming approach is urgently needed for rhLCV titers quantification.

To address this, we developed a real-time quantitative PCR (real-time qPCR)-based approach to quantify the copy numbers of rhLCV by amplifying the rhLCV BALF5 gene, a single-copy gene within the rhLCV genome (Fig. 1F). The full length of BALF5 was cloned into the pCDH vector, which was used to generate a standard curve for subsequent real-time qPCR assay (Fig. 1F). The total rhLCV DNA copy numbers were used to represent the titers of rhLCV. Using this method, we quantified the titers of five independent rhLCV stocks produced by LCL8664. As expected, rhLCV titers decreased with increased dilution fold (Fig. 1G). The titers of these five lots of rhLCV stock solution ranged from 6.8 to 8.6 (log10) per microliter of the viral stock solution (Fig. 1H).

We used this real-time qPCR approach to determine LCV copy numbers in cynomolgus macaques. Virus copy numbers in whole blood and saliva of four cynomolgus macaques were assessed (Fig. 1I and J). Except for cynomolgus macaque #264, the rhLCV copy number remained undetectable in the whole blood and saliva of the other three cynomolgus macaques (Fig. 1I and J). The BALF5 gene sequence of cyLCV (endogenous LCV of cynomolgus macaques) is unknown. Given the high sequence homology between rhLCV and cyLCV, it remains unclear whether the detected BALF5 gene in cynomolgus macaque samples originated from rhLCV or cyLCV. Hence, cynomolgus macaque #264 may be naturally infected by rhLCV or cyLCV. Consistent with real-time qPCR results, EBV gB, gHgL, gp42, and gp350 cross-reactive antibodies were detected in the sera of cynomolgus macaque #264 instead of the other three macaques (Fig. 1K through N). PBMCs of rhLCV or cyLCV-negative cynomolgus macaques #975, #502, and #111 were used for the subsequent experiments to detect the susceptibility of rhLCV infection.

In summary, a real-time qPCR-based approach provides a more time-efficient method to quantify rhLCV titers and determine rhLCV copy numbers in primates to demonstrate infectious state.

### Cynomolgus macaque lymphocytes were susceptible to rhLCV *in vitro*

**Fig 2:**
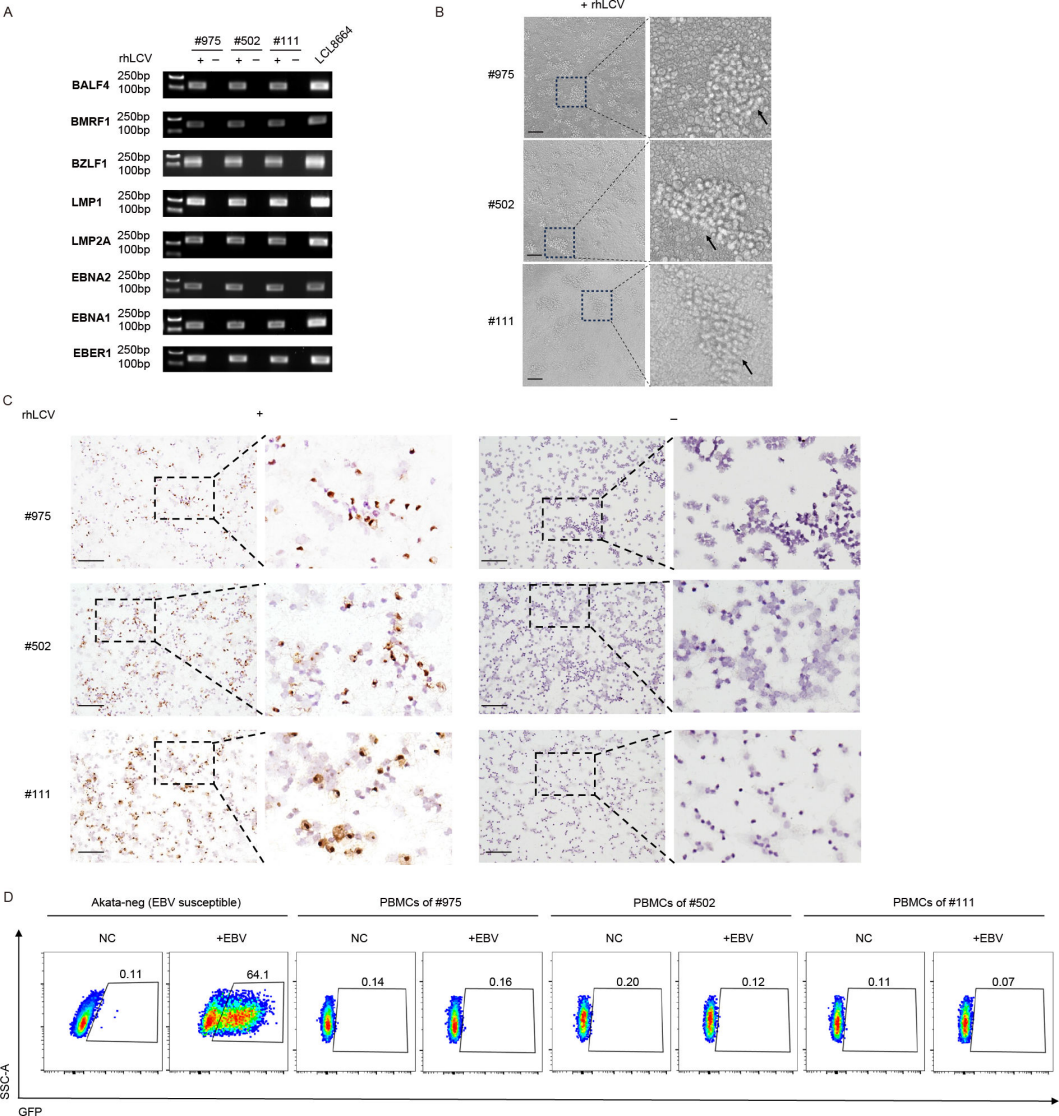
Cynomolgus macaque PBMCs were susceptible to rhLCV infection *in vitro*. (**A**) rhLCV gene expression in PBMCs of cynomolgus macaques #975, #502, and #111 seven days after rhLCV treatment. PBMCs that were not exposed to rhLCV were used as negative controls. LCL8664 was used as a positive control. Primers used in this assay are shown in Fig. 1C. (**B**) Representative images of PBMCs of cynomolgus macaques #975, #502, and #111, 7 days after rhLCV treatment (scale bar = 500 μm). (**C**) Representative images of PBMCs of cynomolgus macaques #975, #502, and #111, 7 days after rhLCV treatment, hybridized for EBER (scale bar = 50 μm). PBMCs that were not exposed to rhLCV were used as a negative control. (**D**) Representative flow cytometry images of PBMCs of cynomolgus macaques #975, #502, and #111 48 h post-exposed to CNE2-EBV-GFP. Akata cells (human B-cell line, EBV negative) that were exposed to EBV were used as positive controls, while PBMCs that were not exposed to EBV were used as negative controls.

Next, we assessed the susceptibility of cynomolgus macaque PBMCs to exogenous LCV, rhLCV, and EBV. 1 × 10^5^ freshly isolated cynomolgus macaque PBMCs were resuspended in 100 μL viral solution containing approximately 1.4 × 10^10^ copies of rhLCV. After 7 days, we assessed the expression of latent and lytic rhLCV genes using nested PCR with uninfected PBMCs as negative controls and LCL8664 as positive control (Fig. 2A). Compared to uninfected controls, rhLCV latent genes, lytic genes, and rhLCV-EBER1 were detected in PBMCs infected by rhLCV (Fig. 2A). The results confirmed that rhLCV successfully infected cynomolgus macaque PBMCs with a gene expression pattern similar to LCL8664 (Fig. 2A). The results showed that EBNA2 and EBNA1 expression levels were relatively low at day 7 post-infection (Fig. 2A). EBNA2 is required for EBV-induced transformation. EBNA2 protein expression peaks at approximately 2 days post-infection and then decreases to a stable level in LCLs (30). EBNA1 plays pivotal roles in EBV replication, genome persistence, and transcription, which is expressed in latency stages I, II, and III (31). However, EBNA1 is not essential for transformation (32). Although the timing of EBNA1 protein peak expression across infection days in primary B cells remains to be determined, the transcript levels are significantly low in all forms of latency (33). Besides, cell aggregation was observed 7 days post-infection (Fig. 2B). To further confirm rhLCV infection, EBER *in situ* hybridization was performed. Seven days post-infection, EBER-positive cells were detected in a subset of PBMCs from the three infected macaques, whereas no EBER signal was observed in the uninfected controls (Fig. 2C).

In contrast, EBV was unable to infect cynomolgus macaque lymphocytes (Fig. 2D). Epithelial cell-derived CNE2-EBV-GFP was used to infect cynomolgus macaque lymphocytes, with Akata cells (human B-cell line) serving as a positive control. When ~64% Akata cells became GFP positive, no GFP-positive cells were detected in cynomolgus macaque lymphocytes exposed to CNE2-EBV-GFP (Fig. 2D).

**Fig 3:**
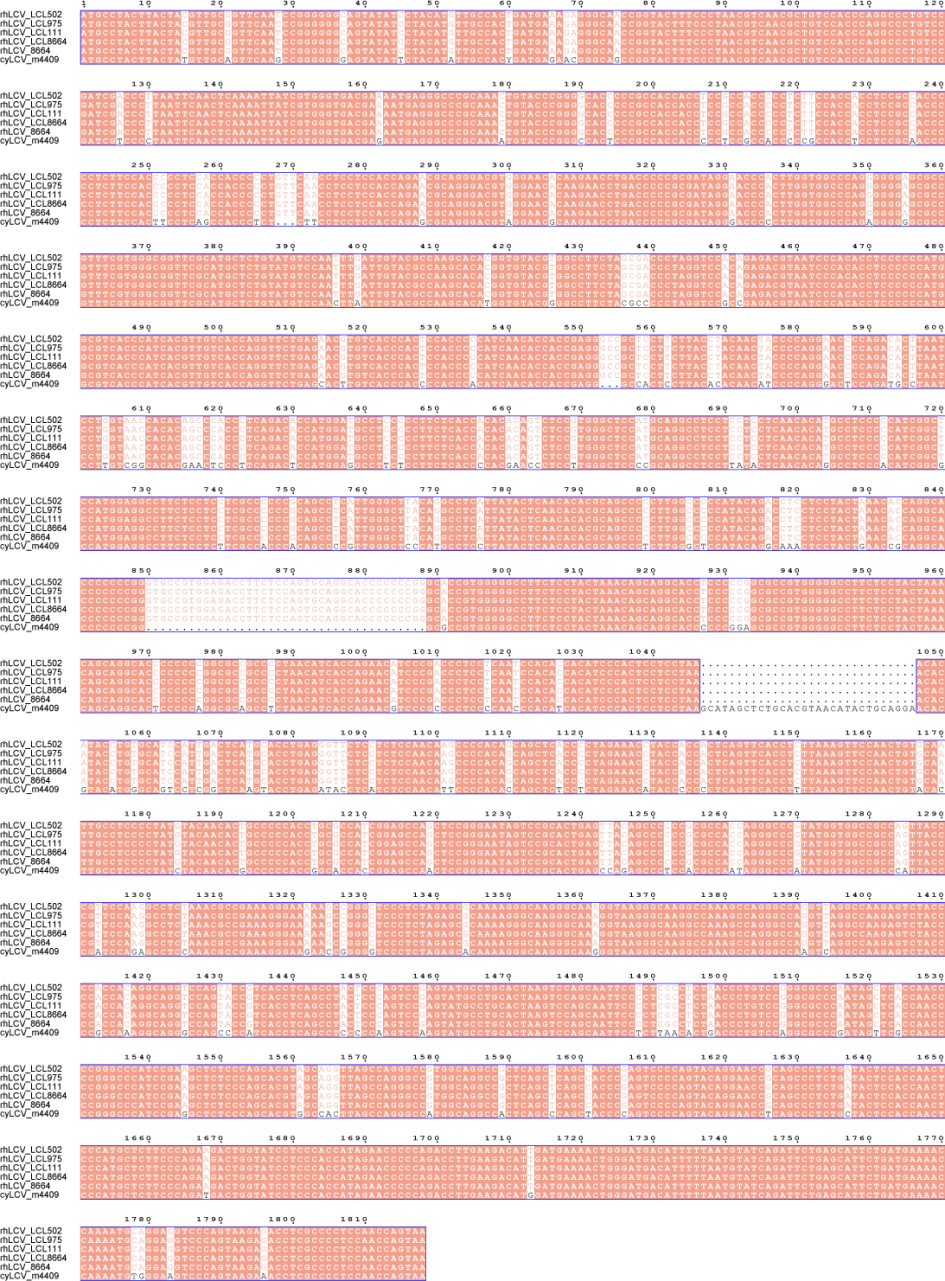
Nucleic acid sequence alignment of EBNA2 of reported rhLCV (8664, GenBank accession number AAK95414), cyLCV (m4409, GenBank accession number KT072774), LCL8664 used in this study, and cy-LCL111, cy-LCL502, as well as cy-LCL975 generated in this study. The sequences were aligned using CLUSTALW and ESPript 3. Conserved sites are highlighted in red boxes with white characters.

Importantly, we sequenced the EBNA-2 sequence of LCL8664 and newly established LCLs to further confirm that newly established LCLs were induced by rhLCV instead of cyLCV. The results demonstrated that LCV in cy-LCL111, cy-LCL502, and cy-LCL975 was rhLCV (Fig. 3). Overall, the findings suggested that cynomolgus macaque lymphocytes are susceptible to rhLCV.

In a word, LCV infects only closely related primates. Cynomolgus macaque lymphocytes are susceptible to rhLCV but not to EBV *in vitro*.

### rhLCV successfully transformed LCLs derived from cynomolgus macaques

After confirming that cynomolgus macaque B cells are susceptible to rhLCV, we next investigated whether cynomolgus macaque B cells can be transformed into LCLs by rhLCV.

**Fig 4:**
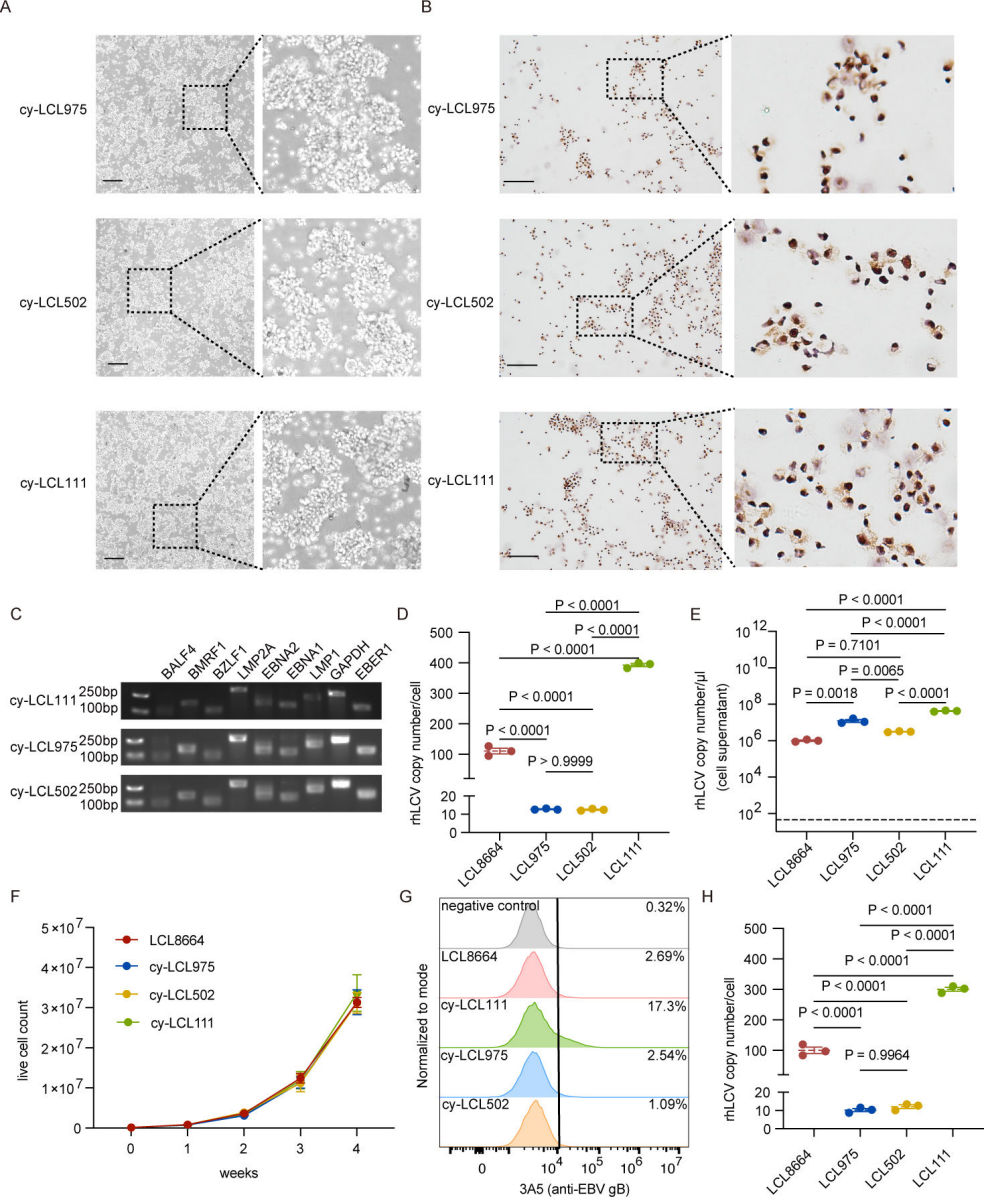
rhLCV-induced immortalization of cynomolgus macaque B cells. (**A**) Representative images of cy-LCL975, cy-LCL502, and cy-LCL111 (scale bar = 500 μm). LCL formation was observed 6 weeks post-infection. (**B**) Representative images of EBER-ISH assay for LCLs 6 weeks post-infection (scale bar = 50 μm). (**C**) rhLCV gene expression profiles in cy-LCL111, cy-LCL975, and cy-LCL502 after 6 weeks of infection. Primers used in this assay are shown in Fig. 1C. (**D**) rhLCV copy numbers in each cell of LCL8664, cy-LCL975, cy-LCL502, and cy-LCL111. cy-LCL975, cy-LCL502, and cy-LCL111 were detected at 6 weeks post-infection. Primers are shown in Fig. 1F. (**E**) rhLCV copy numbers per μL in the undiluted supernatant of LCL8664, cy-LCL975, cy-LCL502, and cy-LCL111 after induced by 20 ng/mL TPA and 3 mM NaB for 12 h at a cell density of 3 × 10^6^ cells/mL. The dashed line indicates the detection limit. Primers are shown in Fig. 1F. (**F**) The growth curve of LCL8664, cy-LCL975, cy-LCL502, and cy-LCL111. cy-LCL975, cy-LCL502, and cy-LCL111 were detected starting at 40 passages (week 0 timepoint). (**G**) rhLCV-gB expression on the surface of LCL8664, cy-LCL975, cy-LCL502, and cy-LCL111. cy-LCL975, cy-LCL502, and cy-LCL111 were detected at 40 passages. (**H**) rhLCV copy numbers in each cell of LCL8664, cy-LCL975, cy-LCL502, and cy-LCL111. cy-LCL975, cy-LCL502, and cy-LCL111 were detected at 40 passages. Primers are shown in Fig. 1F. (**D–F and H**) Data points are shown as the mean ± SEM (*n* = 3). (**D, E, and H**) *P* values calculated using one-way ANOVA with Tukey’s multiple comparison are shown as precise values.

Six weeks after rhLCV infection, LCL formation was observed in PBMCs derived from all three cynomolgus macaques (Fig. 4A). In contrast, PBMCs that were not exposed to rhLCV did not survive beyond 6-week culture. EBER *in situ* hybridization confirmed that almost all cells of each LCL were rhLCV-EBER positive, indicating that the transformed LCLs harbored rhLCV (Fig. 4B). The rhLCV gene expression profiles of cy-LCL111, cy-LCL975, and cy-LCL502 closely resembled those of LCL8664 (Fig. 4C). Latent, immediately early lytic, early lytic, and late lytic genes were expressed, suggesting that spontaneously lytic activity in rhLCV-transformed LCLs derived from cynomolgus macaque (Fig. 4C).

To further characterize these LCLs, we quantified the rhLCV copy numbers per transformed cell. In the LCL8664 cell line, an average of approximately 110 copies of rhLCV were detected per cell (Fig. 4D). Similarly, cy-LCL975 and cy-LCL502 exhibited an average of around 12 copies per cell, while cy-LCL111 showed a significantly higher viral load, with an average of 393 copies per cell (Fig. 4D). Additionally, we assessed rhLCV copy numbers in the cell culture supernatant of LCLs 7 days after induction by TPA and NaB. Among all LCLs, cy-LCL111 produced the highest amount of rhLCV in the supernatant after induction among the LCLs, exceeding 1 × 10^7^ copies per microliter of the supernatant (Fig. 4E). These results suggested that LCLs derived from cynomolgus macaque can produce rhLCV upon induction, with cy-LCL111 exhibiting the highest production efficiency.

The established cy-LCLs were subsequently cultured for 4 months (~40 passages) since they transformed at 6 weeks post-infection. We compared the growth kinetics of cy-LCLs at 40 passages to the LCL8664 cell line and found comparable proliferation rates (Fig. 4F). Moreover, cy-gB expression was detected by anti-EBV gB mAb 3A5 in cy-LCLs maintained for 40 passages, confirming stable viral protein expression in long-term cultures (Fig. 4G). rh-LCV copy numbers were detected in the supernatant of cy-LCLs at 40 passages with similar levels of cy-LCLs at 6 weeks after infection (Fig. 4H). These results indicated that the cy-LCLs were actually stably transformed.

Overall, cynomolgus macaque B cells infected by rhLCV can be transformed to LCLs. Almost all LCLs were rhLCV-EBER positive and can be expanded for rhLCV production. Among them, cy-LCL111 harbored the highest rhLCV copies in each cell, making it a potential candidate for rhLCV production.

### cy-LCL111 showed significantly higher rhLCV production capability

**Fig 5:**
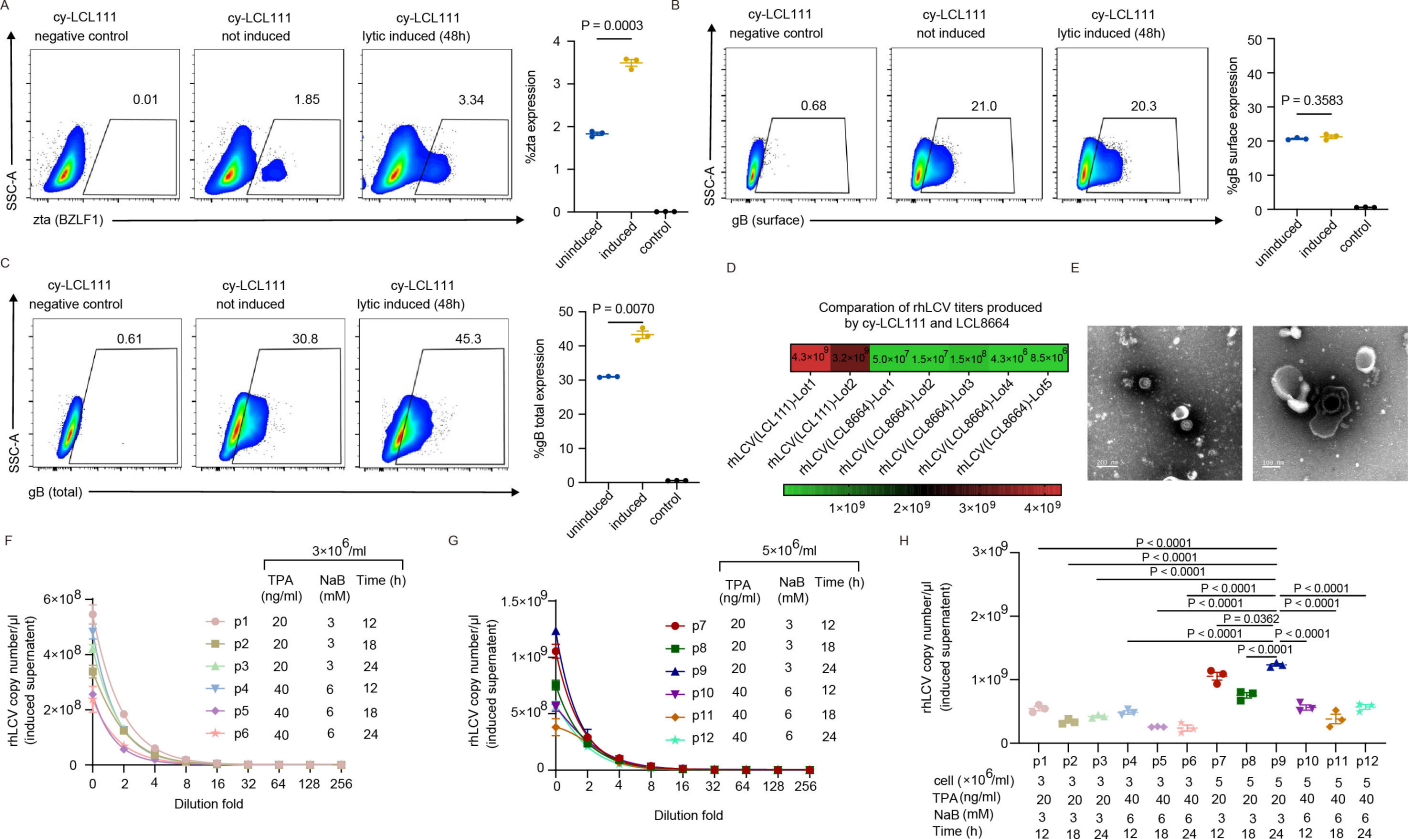
cy-LCL111 exhibited high rhLCV production capacity. (**A**) Percentage of cy-LCL111 cells expressing zta under induced and uninduced conditions. (**B**) The cell surface expression levels of gB in cy-LCL111 under induced and uninduced conditions. (**C**) The cell total expression levels of gB in cy-LCL111 under induced and uninduced conditions. (**D**) Comparison of copy numbers per μL in the viral stock solution of different lots of rhLCV generated by cy-LCL111 or LCL8664. Primers are shown in Fig. 1F. (**E**) Negative-staining TEM images of rhLCV virus generated by cy-LCL111 reveal the presence of viral capsids and viral genome (left panel: scale bar = 200 nm; right panel: scale bar = 100 nm). Due to the preparation procedure for TEM observation, the viral membrane structure was disrupted in the majority of viral particles, preventing its visualization. (**F**) rhLCV copy numbers per μL in the serially diluted supernatant after induction by different concentrations of TPA and NaB for different durations at a cell density of 3 × 10^6^ cells/mL. Primers are shown in Fig. 1F. (**G**) rhLCV copy numbers per μL in the serially diluted supernatant after induction by different concentrations of TPA and NaB for different durations at a cell density of 5 × 10^6^ cells/mL. Primers are shown in Fig. 1F. (**H**) rhLCV copy numbers per μL in the undiluted supernatant after induction by different concentrations of TPA and NaB for different durations at different cell densities. *P* values calculated using one-way ANOVA with Dunnett’s multiple comparison are shown as precise values. Primers are shown in Fig. 1F. (**A–C and F–H**) Data points are shown as the mean ± SEM (*n* = 3). (**A–C**) *P* values calculated using an unpaired two-tailed Welch’s t test are shown as precise values.

We performed flow cytometry to determine the proportion of cy-LCL111 cells undergoing lytic replication. We detected the expression of an immediate early protein rh-BZLF1 (zta) and a late protein, rh-gB, of uninduced and induced (48 h) cy-LCL111 by anti-EBV BZLF1 antibody and anti-EBV gB antibody, respectively. The expression of the immediate early gene zta was detected in cy-LCL111 cells, and TPA/NaB treatment further increased the percentage of zta-expressing cells to 3.34% (Fig. 5A). These results indicate that 1.85% of uninduced cy-LCL111 and 3.34% of induced cy-LCL111 had initiated lytic replication. cy-LCL111 cells maintain a predominantly latent phenotype with a small fraction undergoing spontaneous lytic. In addition, for the uninduced cy-LCL111, 21% of cells showed gB surface expression while 30.8% were gB positive after permeabilization (Fig. 5B and C). The higher percentage of total gB-positive cells indicates that a subset of cells synthesized gB intracellularly. These results suggest that approximately 10% uninduced cy-LCL111 were producing virus, while ~20% of cy-LCL111 were covered by infectious virus. After induction, the gB surface expression level did not change compared to uninduced cells, but the total expression was significantly higher (Fig. 5B and C). Induction enhanced intracellular gB production and increased the proportion of virus-producing cells to approximately 15% (Fig. 5B and C). The difference between gB- and zta-positive cell frequencies may be attributed to the distinct kinetics of immediate-early and late gene expression (34, 35).

To evaluate the stability of rhLCV production in cy-LCL111 across cell passages, we purified rhLCV generated by cy-LCL111 at passage 20 (lot 1) and passage 40 (lot 2). Surprisingly, both lot 1 and lot 2 of rhLCV generated by cy-LCL111 showed significantly higher rhLCV copy numbers per microliter of the viral stock solution compared to all lots of virus generated by LCL8664 (Fig. 5D). The rhLCV copy numbers per microliter of the viral stock from lot 1 and lot 2 of rhLCV generated by cy-LCL111 were 4.3 × 10^9^ and 3.2 × 10^9^, respectively (Fig. 5D). To further characterize the rhLCV generated by cy-LCL111, we also performed TEM analysis. The rhLCV capsid and internal rhLCV genome were observed, and the morphology was similar to EBV as well as rhLCV generated by LCL8664 (Fig. 5E).

Collectively, cy-LCL111 demonstrated stable rhLCV production even after being passed down to 20 generations with relatively high viral production yield.

### Optimization of rhLCV induction conditions

To further optimize the induction conditions for rhLCV production, we systematically evaluated the effects of cell density, TPA, and NaB concentrations, and induction duration.

The results indicated that at a cell density of 3 × 10⁶ cells/mL, treatment with 20 ng/mL TPA and 3 mM NaB for 12 h resulted in the highest rhLCV production compared to other tested conditions (Fig. 5F). However, when the concentrations of TPA and NaB were doubled, rhLCV titers declined, likely due to the cytotoxic effects of these agents (Fig. 5F). These findings suggest that excessive exposure to TPA and NaB may compromise cell health, potentially impairing viral replication and release. Thus, optimizing the balance between chemical inducers and cell viability is critical to maximizing viral yield while minimizing cytotoxicity.

Interestingly, a significant increase in virus yield was observed when the cell density was raised to 5 × 10⁶ cells/mL, suggesting that a higher number of cells in the culture contributed to enhanced viral production (Fig. 5G). Specifically, cy-LCL111 at densities of 3 × 10^6^ cells/mL and 5 × 10^6^ cells/mL induced by 20 ng/mL TPA and 3 mM NaB for 24 h produced 4.22 × 10^8^ and 1.23 × 10^9^ rhLCV copies per microliter of the supernatant, respectively (Fig. 5F–5H). Among all tested conditions, the highest rhLCV yield was achieved when 5 × 10⁶ cells/mL of cy-LCL111 were induced with 20 ng/mL TPA and 3 mM NaB for 24 h, indicating that a prolonged induction period under optimal cell density and chemical stimulation further enhanced viral production (Fig. 5H).

These findings highlight the importance of balancing cell density, chemical inducers, and induction duration to maximize rhLCV yield while minimizing cytotoxic effects, providing valuable insights for optimizing large-scale viral production.

### Infectious and transformation efficiency of rhLCV generated by cy-LCL111

The genome copy numbers of rhLCV generated by cy-LCL111 were relatively high. However, genome copy numbers cannot accurately reflect infectious titers as this approach cannot distinguish infectious and non-infectious virus. In addition, more than one virus per cell is needed for infection to succeed.

**Fig 6:**
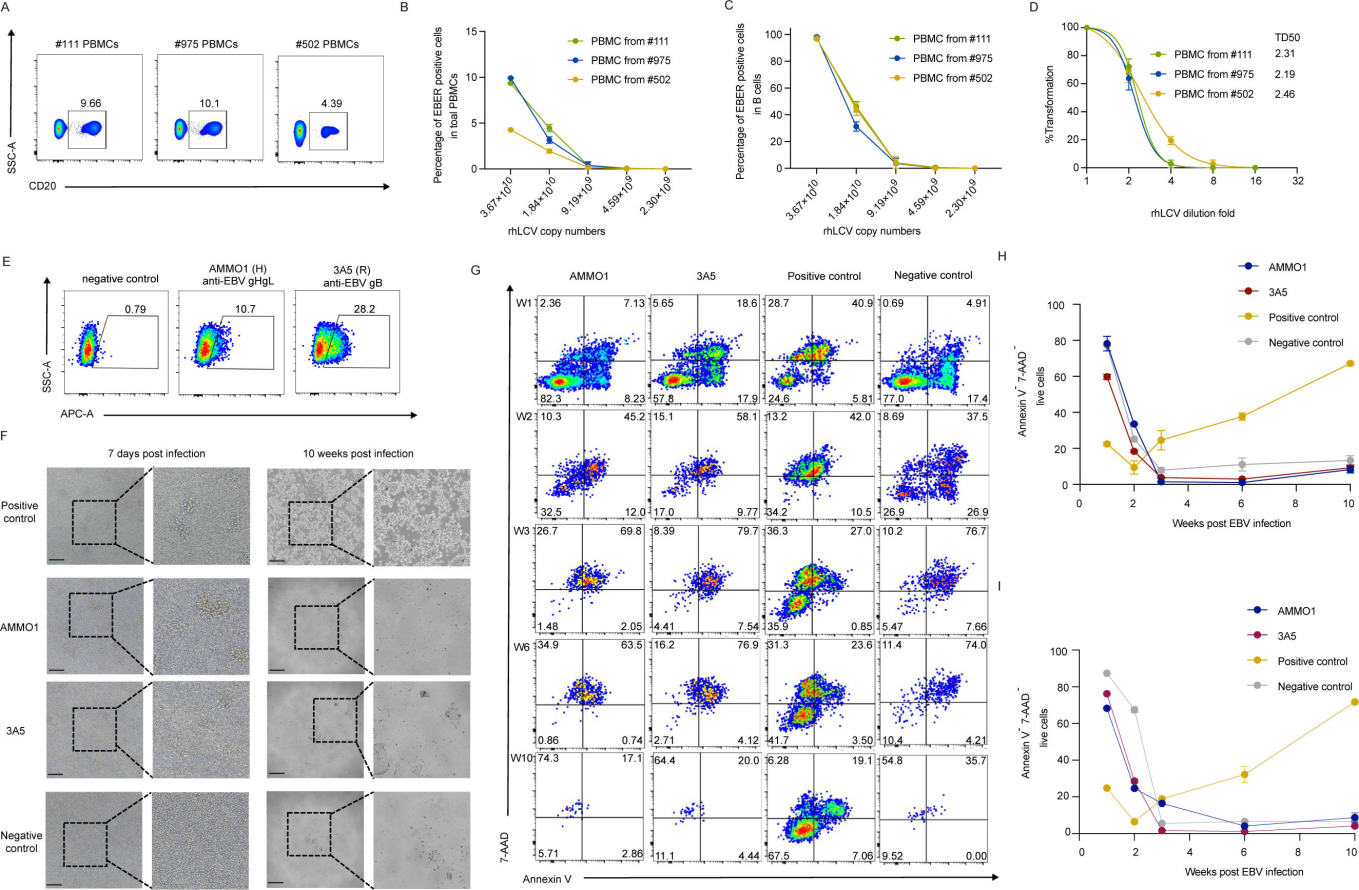
Anti-EBV nAbs inhibited the transformation of rhLCV produced by cy-LCL111. (**A**) Percentage and representative flow images of B cells in three cynomolgus macaque PBMCs (#111, #975, and #502). (**B**) Percentage of EBER^+^ cells in total PBMCs of cynomolgus macaque #111, #975, and #502. EBER^+^ cells were determined by EBER ISH. (**C**) Percentage of EBER^+^ cells in B cells of cynomolgus macaque #111, #975, and #502. EBER^+^ cells were determined by EBER ISH. (**D**) The transformation ability of rhLCV produced by cy-LCL111 was determined at 10 weeks post-infection. The transformation assay was performed in two independent experiments, each with 18 replicates (*n* = 18) per cynomolgus macaque. Data points are shown as the mean ± SEM. Curves were fit using a parameter nonlinear regression. TD50: 50% transforming dilution fold. (**E**) Binding ability of anti-EBV nAbs 3A5 and AMMO1 to cy-LCL111. (**F**) Inhibition of rhLCV-induced LCL formation in PBMCs treated by 3A5 and AMMO1 (scale bar = 500 μm). (**G**) Representative flow cytometry images of PBMCs treated by 3A5 or AMMO1 before rhLCV infection. Live cells were evaluated 1, 2, 3, 6, and 10 weeks post-rhLCV infection by annexin V-7-AAD staining. (**H**) The dynamics of live PBMCs from cynomolgus macaque #111 treated with 3A5 or AMMO1 before rhLCV infection. (**I**) The dynamics of live PBMCs from cynomolgus macaque #502 treated with 3A5 or AMMO1 before rhLCV infection. (**F–I**) PBMCs infected by rhLCV without antibody treatment served as the positive control. Uninfected PBMCs without antibody treatment were used as the negative control. (**B, C, H, and I**) Data points are shown as the mean ± SEM (*n* = 3).

Hence, to determine the infectious efficiency of rhLCV generated by cy-LCL111, we infected PBMCs of cynomolgus macaques with serially diluted rhLCV generated by LCL111 and detected the infected cells by EBER ISH at 2 days post-infection. The percentage of B cells in PBMCs from three cynomolgus macaques #111, #975, and #502 was 9.99%, 10.1%, and 4.39%, respectively (Fig. 6A). Correspondingly, the percentage of EBER^+^ cells among total PBMCs varied due to differences in B-cell frequencies (Fig. 6B). But when normalized to the B-cell population, all three macaques showed comparable susceptibility to rhLCV infection, indicating consistent infectivity of the virus produced by cy-LCL111 (Fig. 6C). Approximately 3.67 × 10^10^ genome copy numbers of rhLCV infected nearly all B cells within 1 × 10⁵ PBMCs (Fig. 6C).

Moreover, we performed the transformation assay to determine the transformation ability of rhLCV produced by cy-LCL111. Virus dilution started at 3.67 × 10^10^ genome copies, and transformation efficiency was assessed at 10 weeks post-infection. The TD50 (50% transforming dilution fold) for cynomolgus macaques #111, #975, and #502 was 2.31, 2.19, and 2.46 (Fig. 6D). The corresponding genome copy numbers for 50% transforming efficiency of each cynomolgus macaque were 1.59 × 10^10^, 1.68 × 10^10^, and 1.49 × 10^10^, respectively.

Together, although the qPCR-based methods cannot distinguish infectious and non-infectious virus, approximately 3.67 × 10^10^ genome copy numbers of rhLCV were required for infection of all B cells within 1 × 10⁵ PBMCs. At least 1.49 × 10^10^ genome copy numbers of rhLCV were sufficient to achieve 50% transformation.

### EBV nAbs cross-react with rh-gB and rh-gHgL to prevent cy-LCL formation induced by rhLCV infection

Given the high homology between rhLCV and EBV, rhLCV infection of rhesus macaques has been widely used as a model to assess the efficiency of EBV vaccines and nAbs (16, 17, 26). Since cynomolgus macaques are also susceptible to rhLCV infection, it is critical to determine whether EBV-specific nAbs can inhibit the rhLCV infection and transformation of cynomolgus macaque PBMCs.

Previous studies have reported that EBV-specific nAbs, including gH/gL-specific nAbs AMMO1 and E1D1 as well as gB-specific nAb 8A9, cross-reacted with rhLCV (16, 17, 36). In this study, we further confirmed that AMMO1 and a gB-specific nAb 3A5 cross-reacted with the newly established rhLCV-positive cell line cy-LCL111, indicating their potential to block the rhLCV infection process (Fig. 6E). The binding percentages of AMMO1 and 3A5 were 10.7% and 28.2%, respectively, which may be attributed to the fact that only a part of rhLCV within cy-LCL111 was in the lytic phase with glycoprotein expression (Fig. 4C and 6E). To further investigate whether these nAbs could block rhLCV infection and subsequent LCL transformation, 200 μg AMMO1 or 3A5 were incubated with approximately 4 × 10^10^ rhLCV copies generated by cy-LCL111 for 3 h before adding to PBMCs isolated from cynomolgus macaque #975, #502, and #111. PBMCs incubated with rhLCV alone served as a positive control, while PBMCs not exposed to either rhLCV or nAbs were used as a negative control. The results demonstrated that both AMMO1 and 3A5 inhibited the LCL transformation, and all treated cells died within 10 weeks post-infection (Fig. 6F). To further confirm the transformation inhibition of nAbs, we performed the live cell analysis of the PBMCs infected by rhLCV with or without 3A5 and AMMO1 treatment by annexin V-7-AAD staining. Non-transformed cells cannot be maintained long-term *ex vivo* and naturally disappear. Almost no live cells can be detected in 3 weeks post-infection, and nearly all cells turned into cell debris in the antibody-treated wells and negative control wells (Fig. 6G through I). In contrast, cells in the positive control wells were transformed into LCLs and continuously proliferated (Fig. 6G through I). These findings indicate that AMMO1 and 3A5 can successfully block rhLCV-induced transformation of cynomolgus macaque PBMCs.

**Fig 7:**
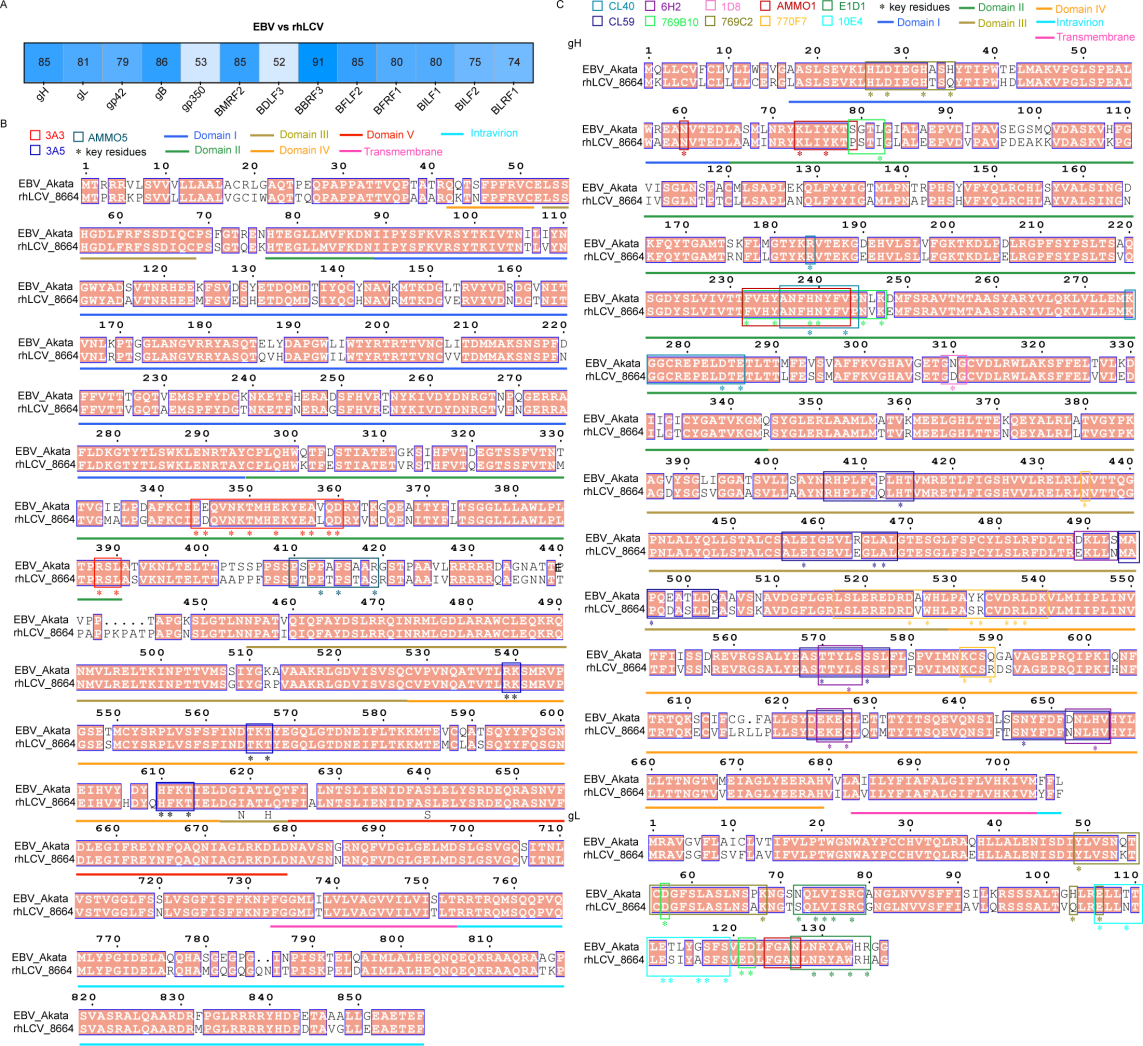
Amino acid sequence alignment of glycoproteins of rhLCV (8664) and EBV (Akata). (**A**) Homology of glycoproteins of rhLCV and EBV. The sequences were aligned using CLUSTALW and ESPript 3. The numbers represent the percentage of homology. (**B**) Amino acid sequence alignment of gB (BALF4) of rhLCV and EBV. (**C**) Amino acid sequence alignment of gH (BXLF2) and gL (BKRF2) of rhLCV and EBV. (**B–C**) The sequences were aligned using CLUSTALW and ESPript 3. Conserved sites are highlighted in red boxes with white characters.

**Fig 8:**
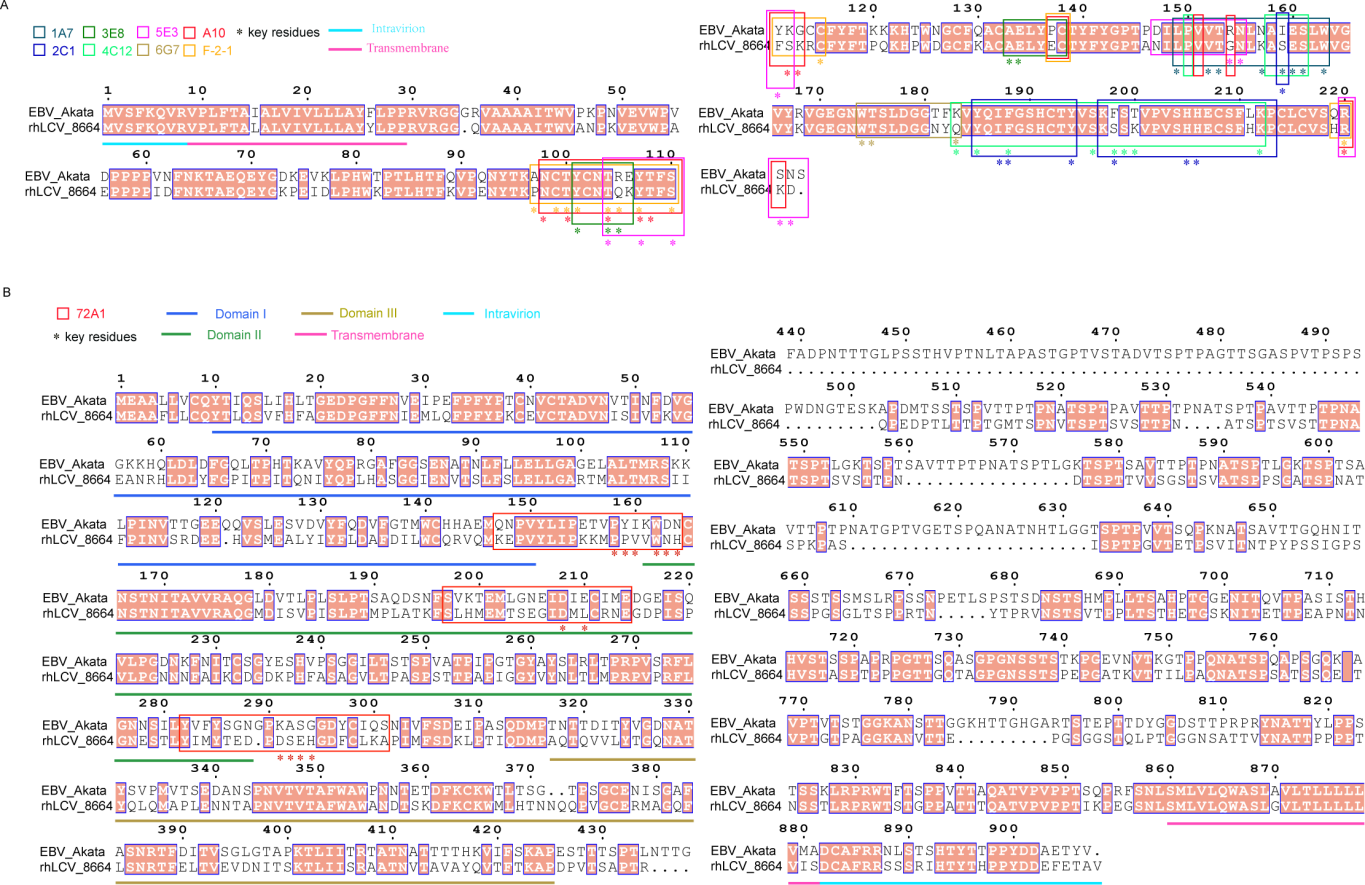
Amino acid sequence alignment of gp42 and gp350 of rhLCV (8664) and EBV (Akata). (**A**) Amino acid sequence alignment of gp42 (BZLF2) of rhLCV and EBV. (**B**) Amino acid sequence alignment of gp350 (BLLF1) of rhLCV and EBV. The sequences were aligned using the CLUSTALW and ESPript 3. Conserved sites are highlighted in red boxes with white characters.

Next, we evaluate the similarity of the amino acid sequence of glycoproteins of rhLCV and EBV. Viral glycoproteins BMRF2, BBRF3, BFLF2, BFRF1, BILF1, BILF2, and BLRF1 all showed high sequence similarity between rhLCV and EBV (Fig. 7A). Notably, the essential entry glycoproteins gH, gL, gp42, and gB exhibited sequence similarities of 85%, 81%, 79%, and 86%, respectively, between EBV and rhLCV (Fig. 7A). More importantly, the epitopes targeted by 3A5 are completely conserved between rhLCV and EBV, enabling its cross-reactivity with cy-LCL111 and its ability to inhibit rhLCV infection (Fig. 7A and B). Similarly, the epitopes of other EBV gB-specific nAbs, AMMO5 and 3A3, also showed high sequence similarity between rhLCV and EBV (Fig. 7B). Moreover, key residues recognized by gHgL-specific antibodies, such as AMMO1, CL40, 1D8, and 6H2 were highly conserved between the two viruses (Fig. 7C). Additionally, the sequence homology of gp42-targeting nAbs was also nearly identical between EBV and rhLCV (Fig. 8A). However, as for gp350, the sequence similarity of gp350 of EBV and rhLCV is relatively low (53%), resulting in the gp350 chimeric rhLCV being needed to evaluate the efficiency of gp350-specific antibodies (Fig. 7A and 8B) (17, 25). These results demonstrated that vulnerable sites of each key entry glycoprotein, gHgL, gp42, and gB remain conserved in LCV evolution. Hence, the efficiency of EBV key entry glycoprotein-specific nAbs or vaccines can be evaluated by the rhLCV infection model.

**Fig 9:**
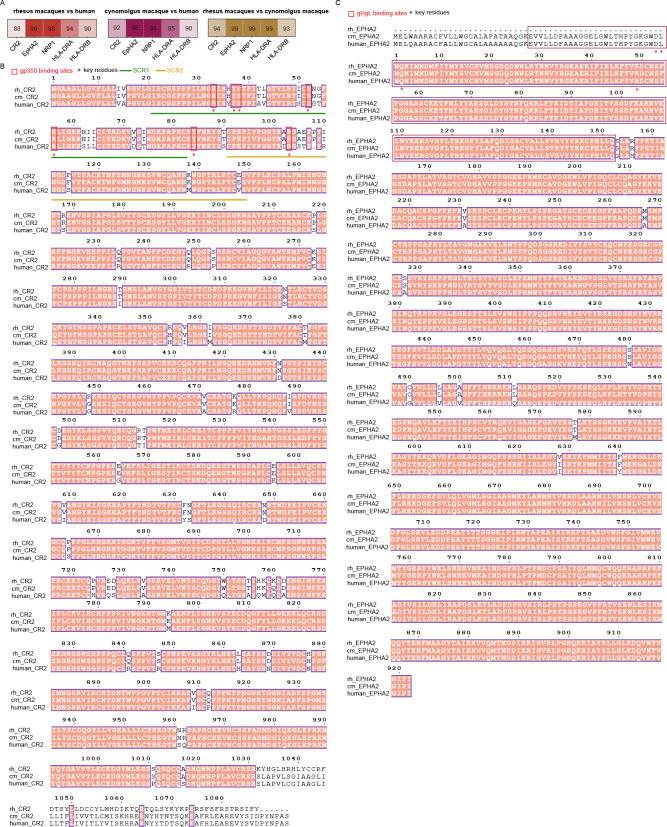
Amino acid sequence alignment of key receptors for EBV infection of human, cynomolgus macaques, and rhesus macaques. (**A**) Homology of receptors for EBV infection. The sequences were aligned using CLUSTALW and ESPript 3. The numbers represent the percentage of homology. (**B**) Amino acid sequence alignment of CR2 of human, rhesus macaque, and cynomolgus macaque. (**C**) Amino acid sequence alignment of EphA2 of human, rhesus macaque, and cynomolgus macaque. (**B and C**) The sequences were aligned using CLUSTALW and ESPript 3. Conserved sites are highlighted in red boxes with white characters.

**Fig 10:**
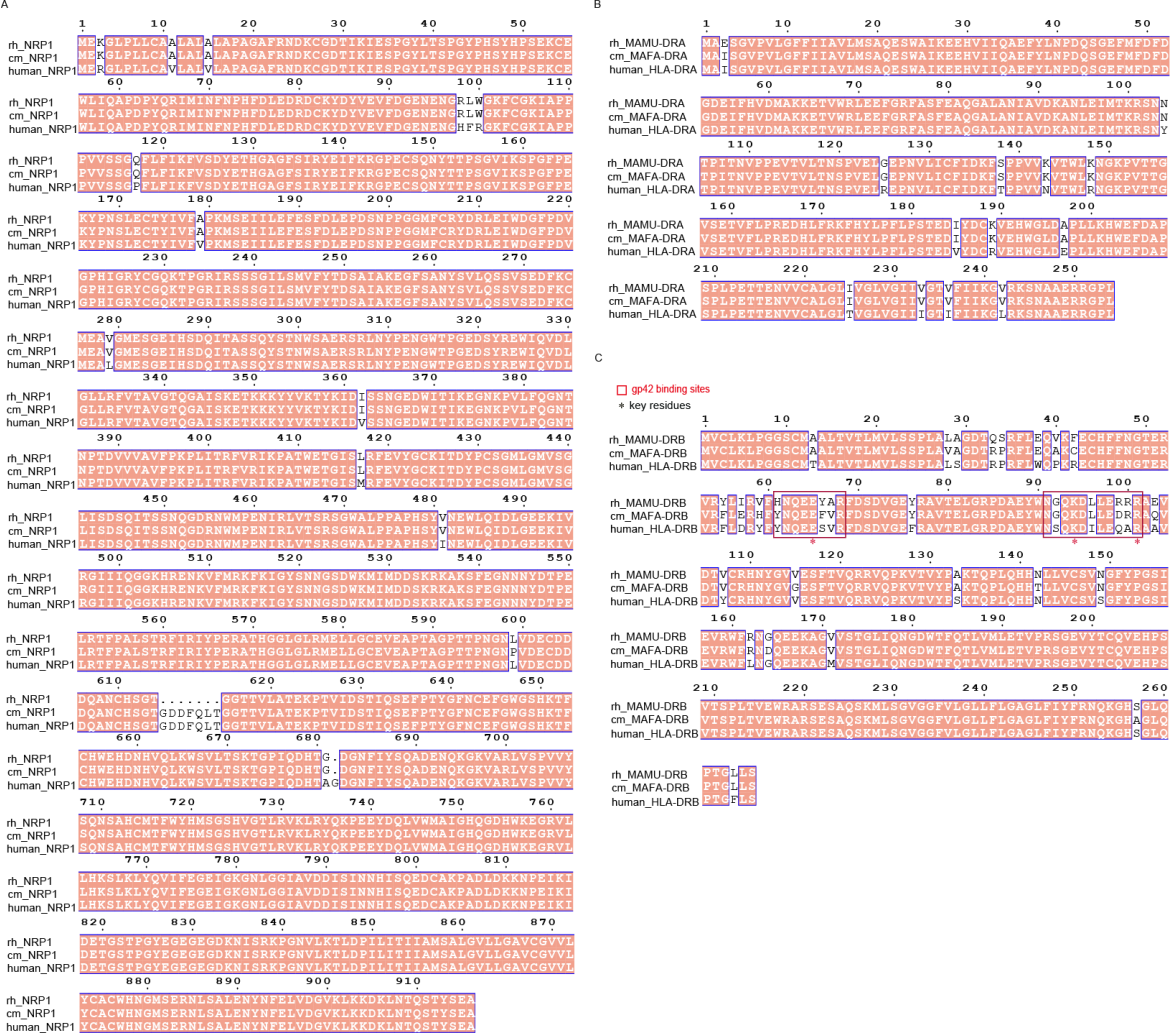
Amino acid sequence alignment of key receptors NRP1, HLA-, MAMU-, or MAFA-DRA and HLA-, MAMU-, or MAFA-DRB for EBV infection of human, cynomolgus macaques, and rhesus macaques. (**A**) Amino acid sequence alignment of NRP1 of human, rhesus macaque, and cynomolgus macaque. (**B**) Amino acid sequence alignment of HLA-DRA of human, MAMU-DRA of rhesus macaque, and MAFA-DRA of cynomolgus macaque. (**C**) Amino acid sequence alignment of HLA-DRB of human, MAMU-DRB of rhesus macaque, and MAFA-DRB of cynomolgus macaque. The sequences were aligned using CLUSTALW and ESPript 3. Conserved sites are highlighted in red boxes with white characters.

We also analyzed the homology of key host receptors for EBV infection, CR2 (37), EphA2 (38, 39), neuropilin-1 (40), and HLA-DR (41), among cynomolgus macaque, rhesus macaques, and humans (Fig. 9 and 10). Compared to humans, the sequence similarities of CR2, EphA2, NRP1, HLA-DRA and HLA-DRB in cynomolgus macaque are 92%, 98%, 98%, 95%, respectively (Fig. 9 and 10). Although LCVs are highly conserved across primates, whether they utilize the same mechanism to infect the host remains to be determined.

In summary, these results demonstrated that EBV-specific nAbs prevent rhLCV-induced transformation of cynomolgus macaque PBMCs *in vitro*. The high sequence conservation of key EBV-targeted glycoproteins in rhLCV further supports the use of rhLCV infection in cynomolgus macaques as a promising preclinical model for evaluating EBV-specific nAbs and vaccine candidates.

## DISCUSSION

In this study, we demonstrated that rhLCV, instead of EBV, can effectively infect PBMCs of cynomolgus macaque, a primate species closely related to rhesus macaque. rhLCV can transform cynomolgus macaque PBMCs into LCLs. At least 1.49 × 10¹⁰ rhLCV genome copies were needed to achieve 50% transformation efficiency. Importantly, the newly established LCL cell line, LCL111, exhibited the highest viral production capacity, which was stable for long-term culture. Moreover, EBV gB-specific nAb 3A5 and gHgL-specific nAb AMMO1 bound to rhLCV-infected cells, indicating cross-reactivity with rh-gB and rh-gHgL, respectively. Notably, both 3A5 and AMMO1 inhibited rhLCV-induced transformation of cynomolgus macaque PBMCs into LCLs. Overall, our study highlights the potential of rhLCV infection in cynomolgus macaques as a valuable surrogate model for EBV-related research.

A significant challenge in EBV-related research is the lack of suitable animal models due to host restrictions, which limits the ability to study viral pathogenesis, immune responses, and vaccine as well as nAbs efficacy. Given the high genetic and functional homology between EBV and rhLCV, rhLCV infection of rhesus macaques has become a widely accepted model for evaluating the efficacy of EBV vaccines and nAbs (16, 17, 25–27). Although LCVs exhibit relatively strict host specificity, LCVs can still infect primates closely related to their natural hosts (42). In this study, we demonstrated that PBMCs from cynomolgus macaque, a species phylogenetically close to rhesus macaques, are susceptible to rhLCV. Six weeks post-rhLCV infection, we observed the successful transformation of cynomolgus macaque PBMCs into LCLs, confirming that rhLCV can drive cellular immortalization in this alternative host. Additionally, both rhLCV latent and lytic genes were expressed in newly established LCLs, similar to the gene expression profiles in LCL8664. Moreover, nearly all cells within the newly formed LCLs were positive for rhLCV-EBER, further supporting the successful transformation induced by rhLCV. Hence, these findings indicated that rhLCV infection of cynomolgus macaques can serve as a surrogate model for EBV-related research, particularly when rhesus macaques are in short supply. However, whether rhLCV can infect cynomolgus macaque through oral challenges *in vivo* needs further investigation. In addition, we could not compare the infectivity between rhesus and cynomolgus macaques because rhLCV-negative rhesus macaques are difficult to obtain.

Given the need for efficient and reproducible viral quantification methods in research, there is an urgent demand for a more rapid and scalable approach to determine rhLCV titers. Previous studies have utilized the LCL transformation assay to quantify rhLCV titers (17, 26). While this method is well established to reflect the infectious ability of rhLCV, it needs a lengthy incubation period of over 6 weeks and requires multiple replicates per viral dilution to achieve reliable results. In this study, we developed a real-time qPCR-based method to quantify the copy number of the rhLCV BALF5 gene as a proxy for rhLCV copy numbers. This approach offers several advantages, including improved efficiency, ease of operation, and a significant reduction in processing time compared to traditional titration methods. Additionally, this method can also be applied to determine the rhLCV copy numbers in the infected primates’ samples. However, a key limitation of this approach is that it did not reflect the titers of virus with infection ability, because it cannot differentiate between infectious and non-infectious viral particles. To address the limitations, engineering rhLCV with a reporter gene, like GFP, provides a more accurate assessment of viral infectivity. Notably, although the qPCR-based method cannot distinguish the infectious virus, we determined that at least 1.49 × 10^10^ genome copy numbers of rhLCV were sufficient to achieve 50% transformation, whereas approximately 3.67 × 10^10^ genome copy numbers of rhLCV infected nearly all B cells within 1 × 10⁵ PBMCs.

The demand for rhLCV in *in vivo* research has increased substantially, highlighting the need to improve its production efficiency. LCL8664 is the commonly used cell line to produce rhLCV, yielding an average of 6.3 × 10^7^ copies per microliter of the viral stock solution. Notably, the newly established cy-LCL111 showed superior and stable rhLCV production capacity throughout prolonged passaging. Moreover, to optimize virus yield, we systematically evaluated 12 different induction conditions in cy-LCL111 cells. Among these, the highest yield of rhLCV was achieved when cy-LCL111 cells at a density of 5 × 10⁶ cells/mL were exposed to 20 ng/mL TPA and 3 mM NaB for 24 h. This finding indicates that both optimal cell density and prolonged chemical induction synergistically enhance viral production. In summary, cy-LCL111 exhibits a greater capacity for rhLCV production, making it a suitable cell line for large-scale virus generation. Furthermore, the improved induction condition protocol in this study provides a valuable framework for future applications requiring high-yield rhLCV production.

EBV infection is a complex process mediated by multiple glycoproteins, including gp350, gHgL, gp42, and gB. The amino acid sequences of key entry glycoproteins gHgL, gB, and gp42 exhibit a high degree of homology between EBV and rhLCV, underscoring their structural and functional similarities. Additionally, several vulnerable sites of gHgL, gB, and gp42 targeted by several EBV-specific nAbs are highly conserved in EBV and rhLCV. In addition to viral glycoproteins, the key cellular receptors of gp42, gHgL, and gB, such as HLA-DR, EphA2, and NRP1, also exhibit strong sequence conservation between humans and cynomolgus macaques. In this study, we demonstrated that EBV gB-specific nAb 3A5 and gHgL-specific nAb AMMO1 cross-reacted with rhLCV glycoproteins and inhibited rhLCV-induced transformation of cynomolgus macaque PBMCs. gHgL and gB, as well as the fusion process, may be conserved among all LCVs. However, the sequence similarity of gp350 between EBV and rhLCV is relatively low. 72A1 cannot neutralize wild-type rhLCV infection of rhesus macaque B cells (25). Hence, Wang et al. developed the chimeric rhLCV to evaluate gp350-based vaccines and gp350-specific nAbs (17, 25). Altogether, these findings provide evidence that rhLCV infection in cynomolgus macaques may serve as a model for evaluating potential interventions targeting the EBV infection process, especially the fusion process. However, serum antibodies induced by the pre-existing cyLCV infection in cynomolgus macaques could inhibit the subsequent rhLCV challenge. Hence, the selection of cyLCV-negative cynomolgus macaques is essential for reliable challenge assays aimed at evaluating the efficiency of vaccine-induced antibodies. The infection status of cynomolgus macaques can be assessed using an enzyme-linked immunosorbent assay (ELISA) based on EBV glycoproteins. Moreover, similar to the rhesus macaques used in EBV-related studies, cynomolgus macaques should be obtained shortly after birth and hand-reared in an LCV-free nursery to prevent natural transmission of endogenous LCV (16, 17, 26).

In conclusion, we demonstrate that cynomolgus macaques are susceptible to rhLCV infection. Notably, we established the rhLCV-induced cy-LCL111 cell line, which exhibited increased viral production capacity. Furthermore, EBV-specific nAbs targeting gHgL and gB inhibited the rhLCV-induced immortalization of cynomolgus macaque B cells. rhLCV infection in cynomolgus macaques may provide a useful surrogate model for EBV-related research.

## MATERIALS AND METHODS

### Cell lines

LCL8664, cy-LCL111, cy-LCL502, and cy-LCL975 were grown in RPMI-1640 medium supplemented with 10% fetal bovine serum (ExCell Bio), 1% GlutaMAX (Invitrogen), 1% sodium pyruvate (Invitrogen), and 1% penicillin-streptomycin (Beyotime) at 37°C in a humidified atmosphere with 5% CO_2_. CNE2-EBV cells (43) were cultured in RPMI 1640 (Invitrogen) with 10% FBS (Invitrogen), antibiotics (penicillin, 100 U/mL; streptomycin, 100 μg/mL; Invitrogen), and maintained under G418 selection (700 μg/mL; MP Biomedicals). Akata cells were cultured in RPMI 1640 (Invitrogen) with 10% FBS (Invitrogen), and antibiotics (penicillin, 100 U/mL; streptomycin, 100 μg/mL; Invitrogen).

### Plasmids

The full length of the sequence of rhLCV BALF5 was amplified by PCR from genomic DNA extracted from LCL8664 and subsequently cloned into the pCDH vector. This plasmid was then utilized to generate a standard curve for the quantification of rhLCV DNA copy numbers.

EBV glycoprotein expression plasmids were constructed as previously reported (8). Briefly, the coding sequences of the ectodomains of gH, gL, gp42, and gB, as well as the N-terminal region of gp350 (gp350N), were PCR-amplified from the EBV M81 strain (GenBank accession no. KF373730.1). Notably, specific residues within gB (WY^112-113^ and WLIW^193-196^) were substituted with homologous residues from HSV-1 (HR^177-178^ and RVEA^258-261^). The gH- and gL-coding sequences were linked via a flexible (GGGGS)₃ linker to facilitate co-expression. The ectodomains of gHgL and gB were cloned into the pCDNA3.1(+) expression vector with a C-terminal 6× His tag, generating pCDNA3.1-gHgL and pCDNA3.1-gB, respectively. Meanwhile, the gp42 and gp350 ectodomains were cloned into the pVRC8400 expression vector, also incorporating a C-terminal 6× His tag, generating pVRC8400-gp42.

### Protein and antibody purification

EBV glycoproteins gHgL, gB, gp42, and gp350, along with EBV-specific nAbs 3A5 and AMMO1, were purified as previously described (8). Briefly, the gHgL, gB, gp42, and gp350N were purified by Ni^2+^ Sepharose 6 Fast Flow beads (GE Healthcare) and subsequently utilized for the ELISA. As for the nAbs purification, 293F cells were transfected with plasmids encoding heavy and light chains of antibody AMMO1 (44) and 3A5 (45). The antibodies were purified via Protein A affinity chromatography (GE Healthcare).

### Animals

Cynomolgus macaques (*Macaca fascicularis*) were housed in the Institute of Zoology, Guangdong Academy of Science. Four 3-year-old female cynomolgus macaques were used for subsequent experiments. The infectious status of these four macaques was determined by real-time qPCR and ELISA.

### EBER *in situ* hybridization

EBER *in situ* hybridization (ISH) detection kit (ZSGB-Bio) was used to detect rhLCV-EBER in rhLCV-infected PBMCs and LCLs according to the manufacturer’s instructions. The stained cells were visualized using an ECLIPSE Ni-U microscopy (NIKON). To determine the EBER titers in the infected PBMCs, the image of each slide was captured by an Automatic Slide Scanner (KFBIO, KF-PRO-020), and the percentages of positive cells were analyzed by ImageJ. The percentage of EBER-positive B cells was calculated by the following formula: number of EBER-positive cells in each slide/(number of total cells in each slide × percentage of CD20^+^ B cells of each macaque) × 100%.

### RNA extraction and PCR assays

Total RNA from 5 × 10^6^ PBMC (rhLCV-infected 7 days) and LCL cells was extracted using a Universal RNA Purification Kit (EZB-RN4), and 1 μg of RNA was reverse transcribed using a 4× Reverse Transcription Master Mix (EZBioscience), following the manufacturer’s instructions.

To evaluate rhLCV infection status and rhLCV gene expression profile, we selected four latent (EBNA1, EBNA2, LMP1, and LMP2A), three lytic (BALF4, BMRF1, and BZLF1) rhLCV genes, and GAPDH (an endogenous control and reference gene for relative quantification) for nested PCR. The primers for nested PCR are shown in Fig. 1C.

EBER1 expression was detected by PCR using the following primers: F: 5′-ctgccctagcggttatgctg-3′; R: 5′-ccagctggtttgaaccgaag-3′.

PCR products were separated by gel electrophoresis and visualized after YeaRed Nucleic Acid Gel Stain (Yeasen) staining.

### rhLCV production

rhLCV was produced as previously described (26). LCL8664, cy-LCL111, cy-LCL502, or cy-LCL975 were resuspended at a density of 3 × 10^6^ cells/mL and treated with 20 ng/mL 12-O-tetradecanoyl-phorbol-13-acetate (TPA; Beyotime) and 3 mM sodium butyrate (NaB; Sigma Aldrich) for 12 h. Then the medium was changed to RPMI complete medium, and cells were cultured for 6 days. Cell supernatants were harvested and filtered through 0.45 μm membrane filter (Millipore, Billerica, MA). Viruses were concentrated by centrifugation at 50,000 × *g* for 2.5 h and resuspended in serum-free RPMI 1640. Viruses were stored at −80°C until use.

### Negative-staining TEM

rhLCV virions were visualized using negative staining TEM. Briefly, viral samples were applied onto 200-mesh carbon-coated copper grids and incubated for 5 min to allow adherence. Excess liquid was carefully removed, followed by two washes with double-distilled water. The grids were then stained for 30 s with freshly filtered 1.6% phosphotungstic acid (pH 6.5). TEM imaging was performed using an FEI Tecnai T12 microscope (FEI, USA) operated at an accelerating voltage of 120 kV. Micrographs were captured at magnifications of 150,000× to ensure detailed structural visualization of the virions.

### DNA extraction and rhLCV copy numbers

DNA was extracted from 250 μL peripheral blood of cynomolgus macaques, 250 μL saliva of cynomolgus macaques, 250 μL of cell culture supernatant, 250 μL of different lots of rhLCV stock solution, or 5 × 10^6^ cells of LCLs using DNA extraction kits (Omega) according to the manufacturer’s instructions. The extracted DNA was subsequently utilized for real-time qPCR to determine rhLCV copy numbers. rhLCV DNA copy numbers were measured by real-time polymerase chain reaction (qPCR SYBR Green Master Mix; Yeasen) using primers specific for rhLCV-BALF5 (F: 5′-cctgcgtctcattcccaagt-3′; R: 5′-accgtctccaggatgtcgta-3′) and amplified for 40 cycles. The DNA copy numbers were calculated based on a standard curve generated from serial dilutions of the pCDH-BALF5 plasmid. The average copy number per cell was calculated using the following formula: rhLCV copy numbers of the total cells/total cell numbers used for DNA extraction.

### ELISA

Serum samples were obtained by centrifuging whole blood at 500 × *g* for 10 min at 4°C. Sera were heat-inactivated by incubation at 56°C for 30 min. ELISA plates (Corning) were pre-coated with 100 ng of purified gp350, gHgL, gB, or gp42 per well and incubated at 37°C for 2 h. After being washed by PBST, the plates were blocked. Serial dilutions of serum samples of cynomolgus macaques (starting at 1:100) were added to the wells and incubated at 37°C for 1 h. After five washes with PBST, wells were treated with horseradish peroxidase-conjugated goat anti-monkey IgG (Invitrogen) diluted at 1:5,000. Signal detection was performed using the EL-TMB kit (Sangon Biotech), and absorbance was measured at 450 nm and 630 nm using a Bio-Tek EPOCH microplate reader.

### PBMC isolation

Ten milliliters of blood was collected into EDTA-coated vacutainers from cynomolgus macaques’ femoral vein. Whole blood was centrifuged at 500 × *g* for 10 min to separate plasma. PBMCs were isolated by Ficoll-Paque density gradient centrifugation according to the manufacturer’s instructions.

### rhLCV infection and B-cell immortalization

Cynomolgus macaque PBMCs were resuspended with 100 μL rhLCV stock solution and seeded in 96-well plates at a density of 1 × 10^5^ cells/well. 3 hours post-incubation, 100 μL of fresh complete medium with 1% GlutaMax and 1% sodium pyruvate was added. After 48 h, the medium was half-changed with fresh complete medium with 1% GlutaMax, 1% sodium pyruvate, and 0.5 μg/mL cyclosporin A. Next, a half-medium change was performed every 7 days by fresh complete medium with 1% GlutaMax, 1% sodium pyruvate, and 0.5 μg/mL cyclosporin A. LCL formation was observed 5–6 weeks post-infection. The nucleic acid sequence of rhLCV-EBNA-2 in LCL8664, cy-LCL111, cy-LCL502, and cy-LCL975 was sequenced.

### Growth curve

The LCL8664 and cy-LCLs, cy-LCL111, cy-LCL502, and cy-LCL975 at 40 passages were seeded in a 48-well plate at 1.5 × 10^5^ cells per well. Cells were passaged every 3–4 days, and live cell numbers were counted weekly.

### EBV production and EBV infection

EBV-GFP derived from CNE2-EBV cells was purified as previously described (8). Briefly, CNE2-EBV cells were induced with 20 ng/mL TPA (Beyotime) and 2.5 mM NaB (Sigma Aldrich) for 12 h, followed by a medium change to fresh complete medium. After 72 h of culture, the supernatant was collected, centrifuged, and filtered through a 0.45 μm membrane. Viruses were then concentrated 100-fold by ultracentrifugation at 50,000 × *g* for 2.5 h, resuspended in serum-free RPMI 1640, and stored at −80°C.

Cynomolgus macaque PBMCs or Akata cells (1 × 10^5^) were seeded in a 96-well plate. EBV-GFP virus (100 μL) was added to each well and cultured for 48 h. Next, cells in each well were centrifuged, washed twice with PBS, and resuspended in 200 μL PBS. GFP-positive-infected cells were quantified by a CytoFLEX LX (Beckman Coulter) and analyzed with FlowJo software X 10.0.7 (Tree Star).

### Optimization of rhLCV production conditions

To further enhance the rhLCV production efficiency, we tested 12 conditions based on the reported rhLCV induction condition described above. Specifically, cy-LCL111 was induced by 20 ng/mL TPA and 3 mM NaB for 12 h (P1), 18 h (P2), and 24 h (P3) or 40 ng/mL TPA and 6 mM NaB for 12 h (P4), 18 h (P5), and 24 h (P6) at the cell density of 3 × 10^6^ cells/mL. Besides, cy-LCL111 density was increased to 5 × 10^6^ cells/mL, and cells were induced by 20 ng/mL TPA and 3 mM NaB for 12 h (P7), 18 h (P8), and 24 h (P9) or 40 ng/mL TPA and 6 mM NaB for 12 h (P10), 18 h (P11), and 24 h (P12). Then the medium was changed to fresh complete medium, and cells were cultured for 6 days. Cell supernatants were centrifuged at 12,000 rpm for 1 h at 4°C and filtered through 0.45 μm membrane filter (Millipore, Billerica, MA). Then, DNA was extracted from 250 μL of supernatant to detect the copy number of rhLCV.

### Cross-reactivity of EBV nAbs to rhLCV-infected LCL

Cells (5 × 10^5^ uninduced or 48 h post-induction cy-LCL111, uninduced cy-LCL975, and uninduced cy-LCL502) were collected and washed twice with PBS. Next, cy-LCL111 was incubated with 0.5 μg 3A5 and AMMO1 at 4°C for 30 min, respectively. cy-LCL975 and uninduced cy-LCL502 were incubated with 0.5 μg 3A5 at 4°C for 30 min. Cells were washed twice with PBS before being incubated with 1:500 diluted goat anti-rabbit IgG Alexa Fluor 647 antibody (Invitrogen) or 1:500 diluted goat anti-human IgG Alexa Fluor 647 antibody (Invitrogen) at 4°C for 30 min.

For intracellular staining, 5 × 10^5^ uninduced or 48 h post-induction cy-LCL111 cells were collected and washed twice with PBS. Single-cell suspensions were incubated with anti-human FcR block (BioLegend) for 30 min at 4°C, fixed and permeabilized with eBioscience Foxp3/Transcription Factor Staining Buffer Set (ThermoFisher), and stained with 0.5 μg 3A5 or anti-BZLF1 (Santa Cruz Biotechnology) for 30 min at room temperature. After three washes, cells were incubated with goat anti-rabbit IgG Alexa Fluor 647 (1:500, Invitrogen) or goat anti-mouse IgG Alexa Fluor 647 (1:500, Invitrogen) secondary antibodies for 30 min at room temperature.

Cells incubated without primary antibodies were used as the negative control. The AF647-positive cells were quantified by CytoFLEX LX (Beckman Coulter), and the data were analyzed by FlowJo software (Tree Star).

### CD20 staining

Cynomolgus macaque PBMCs from each well infected by rhLCV produced by cy-LCL111 at 2 days post-infection were collected and washed twice with PBS. Then, cells were incubated with anti-human CD20-PE (Biolegend) antibodies for 30 min at 4°C. Next, cells were washed with PBS three times, and the percentage of B cells in cynomolgus macaque PBMCs was determined by CytoFLEX LX Flow Cytometer (Beckman Coulter) and analyzed with FlowJo software X 10.0.7 (Tree Star).

### LCL transformation assay

Cynomolgus macaque PBMCs were resuspended with 100 μL rhLCV stock solution produced by cy-LCL111 and seeded in 96-well plates at a density of 1 × 10^5^ cells/well. Three hours post-incubation, 100 μL of fresh complete medium with 1% GlutaMax and 1% sodium pyruvate were added. After 48 h, the medium was half-changed with fresh complete medium with 1% GlutaMax, 1% sodium pyruvate, and 0.5 μg/mL cyclosporin A. Next, a half-medium change was performed every 7 days by fresh complete medium with 1% GlutaMax, 1% sodium pyruvate, and 0.5 μg/mL cyclosporin A. Transformation efficiency was assessed at 10 weeks post-infection. The transformation assay was performed in two independent experiments, each with 18 replicates (*n* =18) per cynomolgus macaque.

### Inhibition of LCL transformation

Fifty microliters AMMO1 (1 mg/mL) or 3A5 (1 mg/mL) was mixed with 50 μL rhLCV viral solution at 37°C for 3 h. Then, 1 × 10^5^ PBMCs were resuspended in the mixture, and 3 h post-incubation, 100 μL fresh complete medium with 1% GlutaMax and 1% sodium pyruvate was added to each well. After 48 h, the medium was half-changed by fresh complete medium with 1% GlutaMax, 1% sodium pyruvate, and 0.5 μg/mL cyclosporin A. Next, a half-medium change was performed every 7 days by fresh complete medium with 1% GlutaMax, 1% sodium pyruvate, and 0.5 μg/mL cyclosporin A. PBMCs incubated with rhLCV alone served as positive controls, while PBMCs incubated with neither rhLCV nor nAbs were used as negative controls.

To test the dynamics of live cells after antibody treatment, PBMCs at 1, 2, 3, 6, and 10 weeks post-infection were stained by APC Annexin V Apoptosis Detection Kit with 7-AAD (Biolegend) following the manufacturer’s instructions.

### Homology analysis

The amino acid sequences of 13 envelope glycoproteins of EBV Akata stain (GenBank accession number KC207813.1) or rhLCV 8664 strain (GenBank accession number AY037858.1) were aligned by CLUSTALW and ESPript 3. Besides, the nucleic acid sequence of EBNA2 of reported rhLCV (8664, GenBank accession number AAK95414), cyLCV (m4409, GenBank accession number KT072774), LCL8664 used in this study, and cy-LCL111 and cy-LCL502, as well as cy-LCL975, generated in this study were aligned by CLUSTALW and ESPript 3. Additionally, the amino acid sequences of CR2 (GenBank accession numbers for human, cynomolgus macaques, and rhesus macaques were NP_001006659, XP_005540745 and XP_077861232.1, respectively), HLA-DRA (GenBank accession numbers for human, cynomolgus macaques, and rhesus macaques were NP_061984, NP_001270278, and XP_014991398, respectively), HLA-DRB (GenBank accession numbers for human, cynomolgus macaques, and rhesus macaques were NP_002115, XP_065400266.1, and XP_028702449, respectively), EphA2 (GenBank accession numbers for human, cynomolgus macaques, and rhesus macaques were NP_004422, XP_045235724, and NP_001035768, respectively) and NRP1 (GenBank accession numbers for human, cynomolgus macaques, and rhesus macaques were XP_006717584, XP_005564992, and XP_015002218.2, respectively) were aligned by CLUSTALW and ESPript 3.

### Statistical analysis

Statistical analyses were performed using GraphPad Prism 8 Software (GraphPad Software, San Diego, CA). Statistical test *n* and *P* values are indicated in figure legends.

## Data Availability

All data supporting the findings of this study are included in the figures.
